# Enhancing assistive technology knowledge through WHO's training in assistive products (TAP): a pre-post quasi-experimental study in Italian undergraduate healthcare education

**DOI:** 10.3389/fresc.2025.1722059

**Published:** 2026-01-29

**Authors:** Gessica Della Bella, Donatella Valente, Justine Gosling, Marco Tofani

**Affiliations:** 1 Management and Diagnostic Innovations & Clinical Pathways Research Area, Neurorehabilitation and Adapted Physical Activity Day Hospital, Bambino Gesù Children’s Hospital IRCCS, Rome, Italy; 2 Department of Human Neurosciences, Sapienza University of Rome, Rome, Italy; 3 Biomedical and Clinical Exercise Science Research Theme, University of Derby, Derby, United Kingdom; 4 Department of Life Sciences, Health and Healthcare Professions, Link Campus University, Rome, Italy

**Keywords:** assistive products, assistive technology, healthcare professionals, nursing, rehabilitation, self-help device, TAP, training

## Abstract

**Background:**

Assistive products (APs) are essential for maintaining or improving individuals’ functioning and independence, yet an estimated 2.5 billion people worldwide require at least one AP. The World Health Organization's Training in Assistive Products (TAP) offers modular, competency-based e-learning to equip healthcare and community workers with the skills to select, fit, use, and follow up on APs.

**Objective:**

To evaluate the effectiveness of selected TAP modules in enhancing knowledge of APs among Italian undergraduate healthcare students in physiotherapy (PT), neuro- and psychomotor therapy of developmental age (TNPEE), and nursing.

**Methods:**

138 students from two universities in Rome completed 20 h of TAP-based training, comprising theoretical lectures and practical laboratories, across three modules: Introduction to Assistive Products, Mobility Assistive Devices, and Transfer Boards. Knowledge was assessed via a 56-item multiple-choice test administered pre- and post-training. Score distributions were tested for normality (Shapiro–Wilk), and pre-post differences were analyzed with Wilcoxon signed-rank tests (effect size r). Between-group comparisons used Bonferroni-adjusted Mann–Whitney U tests.

**Results:**

Of 138 eligible students, 105 completed the Introduction, 95 the Mobility module, and 99 completed the Transfer modules. All modules produced significant knowledge gains (*p* < 0.01) with large effect sizes (r = 0.59–0.79). TNPEE and nursing students showed very large improvements in the Introduction module (r = 0.81 and 0.75; *p* < 0.01), whereas PT students improved non-significantly (r = 0.34; *p* = 0.16), reflecting higher baseline familiarity. PT and TNPEE cohorts achieved the greatest gains in Mobility (r = 0.72–0.80; *p* < 0.01), while Nursing improvements were more modest (r = 0.45; *p* = 0.05). Transfer-Boards training yielded uniformly large gains across all student groups (r = 0.58–0.88; *p* ≤ 0.01).

**Conclusions:**

TAP modules are feasible and effective for undergraduate healthcare curricula, substantially enhancing AP knowledge across the 3 participating professions. Integrating TAP, aligned with WHO's GATE “5P” framework, into existing undergraduate programs can standardize assistive-technology education, reduce discipline-driven competency gaps, and contribute to Universal Health Coverage by preparing a workforce skilled in safe, evidence-based AP provision.

## Introduction

Assistive Technology (AT) is an umbrella term for Assistive Products (APs) and their related systems and services; APs are defined by the World Health Organization (WHO) as any external product – including devices, equipment, instruments or software – whose primary purpose is to maintain or improve an individual's functioning and independence, thereby promoting well-being (1). These products range from basic aids such as spectacles and pill organizers to complex mobility devices like wheelchairs and prostheses, as well as communication aids and digital applications. Despite their critical role in enabling participation and quality of life, an estimated 2.5 billion people currently require at least one assistive product – a figure projected to rise to 3.5 billion by 2050 as populations age and noncommunicable diseases proliferate (2). Yet access remains starkly inequitable: only around 10% of those in low-income countries can obtain the assistive products they need, compared with more than 90% in high-income settings (2, 3). This unmet need presents a profound barrier to Universal Health Coverage and the Sustainable Development Goals (4).

In response to the global access gap, WHO and UNICEF published the Global Report on Assistive Technology in May 2022 (2), which presents a comprehensive analysis of current AT provision and outlines ten key recommendations to strengthen systems, including the development of workforce training programs and integration of assistive products within health policies. Among its strategic imperatives, the report highlights the necessity of standardized, competency-based training for healthcare and community workers to ensure safe and effective selection, fitting, use and follow-up of assistive products.

The WHO's Global Cooperation on Assistive Technology (GATE) initiative operationalizes this vision through a systems-based “5P” framework – People, Policy, Products, Provision and Personnel – designed to address all dimensions of AT access (2, 5). *People* underscores the centrality of users' rights, needs and participation (6); *Policy* calls for embedding APs within national health and social protection policies (7); Products involves ensuring quality (8), affordable items via mechanisms such as the WHO Priority Assistive Products List; *Provision* refers to strengthening service delivery and supply chains (9); and *Personnel* focuses on building and sustaining a trained workforce capable of prescribing, fitting, maintaining and monitoring APs (10). By coordinating interventions across these domains, the 5P model seeks to create resilient AT ecosystems.

Parallel to GATE's emphasis on APs, WHO's Rehabilitation 2030: A Call for Action (11–13) initiative draws attention to a global rehabilitation workforce crisis, noting that up to one-third of the world's population may benefit from rehabilitation services but far too few professionals are equipped to deliver them (14, 15). Available evidence shows that the density of physiotherapists (PT) varies greatly across the region and is largely insufficient to meet population needs. Per 100,000 of the population, PT densities range from 2 in central Asia to 79 in southern Europe, 125 in northern Europe and 179 in western Europe (16) Many low- and middle-income countries report fewer than ten qualified rehabilitation practitioners (e.g., PT, occupational therapists, prosthetics and orthotics technicians) per million inhabitants (15). Workforce shortages are driven by underinvestment in education, low remuneration, burnout, and uneven geographic distribution—factors exacerbated by demographic shifts and the rising burden of chronic conditions (17, 18). This scarcity of skilled personnel directly limits the capacity to deliver APs safely and effectively.

Manship and colleagues recent systematic review (19) highlights a striking global deficit in both the availability of APs and the training required to use them effectively. Across disciplines and regions, practitioners report inconsistent, often cursory coverage of APs in professional curricula, leading to wide knowledge gaps in assessment, prescription, fitting and follow-up. Training offerings are highly variable in depth, duration and modality – with many programs lacking hands-on components or ongoing mentorship – undermining clinicians' confidence and competence in recommending and supporting assistive products (19).

In Italy, the first nationwide rapid Assistive Technology Assessment (rATA) survey, conducted between June and September 2021 on a representative sample of 10,167 citizens, revealed that 52.6% of the population need at least one assistive product, yet 6.7% of these needs remain unmet (20, 21). Need and use were significantly higher among older adults (aged 60 years and above) and those reporting more severe functional limitations, underscoring both demographic and health-related predictors of APs reliance. The study also highlighted the growing role of mainstream digital devices, such as smartphones, which are widely used informally as assistive tools but often fall outside formal funding and training frameworks. Regional disparities in service provision further compound access inequities, calling for targeted policy reforms, enhanced funding mechanisms, and workforce development tailored to Italy's decentralized health system.

To address these gaps, WHO has launched Training in Assistive Products (TAP), an open-access, modular e-learning program developed in collaboration with global partners. TAP comprises a series of modules covering key APs drawn from the WHO Priority Assistive Products List (22), and is structured around four core service steps to build practical competencies, namely Select, Fit, Use and Follow-up. The platform is designed for a broad audience, including primary healthcare workers, community providers, service managers, educators and end-users, and is available in multiple languages, making it adaptable to diverse contexts.

Given Italy's documented high prevalence of APs needs (21), persistent access barriers, and shortage of trained rehabilitation professionals (23), it is imperative to evaluate the effectiveness of WHO's TAP modules within Italian undergraduate healthcare curricula. The present exploratory study aims to assess whether participation in TAP improves undergraduate PT, Nurse and TNPEE students' knowledge and intended practice regarding APs. Findings will inform strategies for strengthening the assistive technology workforce and contribute to the global evidence base on capacity-building interventions in developing assistive technology ecosystems.

## Methods

This Pre-Post Quasi-Experimental study was carried out between May 2024 and June 2025 at two Italian academic institutions - Link Campus University and Sapienza University of Rome - in collaboration with the Bambino Gesù Children's Hospital IRCCS. Given the non-interventional nature of the educational evaluation, formal review by a dedicated ethics committee was waived; nevertheless, all procedures adhered to institutional ethical standards and the principles of the Declaration of Helsinki. Ethical oversight and confirmation of compliance were provided by the Scientific Directorate of Bambino Gesù Children's Hospital in September 2025.

### Participants

All students enrolled in the relevant undergraduate healthcare-professional programs at the two participating universities were eligible to participate, as the TAP training was integrated directly into existing curricular courses. Participation therefore derived from their regular academic enrollment rather than from an external recruitment process. This included 35 students of Physiotherapy (PT) BSc at Link Campus University, 30 students of Neuro- and Psychomotor Therapy of Developmental Age (TNPEE) BSc program, 40 students in the PT BSc and 33 Nursing BSc students at Sapienza University of Rome, yielding a total sample of 138. Prior to data collection, participants received detailed information regarding study objectives and procedures, and provided written informed consent for anonymized use of their data in aggregate form for research dissemination.

### Training: topics and methodology

The TAP-based training (24) was integrated into existing in-person curricular activities at the two participating universities. It is important to note that TNPEE and PT students were in their first academic year, whereas nursing students were in their second. For rehabilitation-focused students at both institutions (TNPEE, PT), TAP modules were delivered during the “Health and Safety Promotion” course in the second semester of the first academic year. For nursing students, TAP was embedded within the “Nursing for Chronicity and Disability” lecture in the second semester of the second year. Each course comprised a total of 20 instructional hours, divided into 4 h of preparatory content, 10 h of TAP-specific modules, and 6 h of hands-on laboratory activities.

Based upon WHO recommendations and the Italian epidemiological profile, needs for rehabilitation (25) and academic educational objectives, the following TAP modules were selected: 1) Introduction to Assistive Products; 2) Mobility Assistive Products; 3) Walking Aids; 4) Transfer Boards. While WHO estimates that the online modules of the selected TAP require approximately 10 h (24), we adapted for our training program, as follow:

1. Introduction to Disability and the International Classification on Functioning, Disability and Health (4 h), delivered as preparatory content and distinct from TAP;
2. Introduction to APs: What are assistive products?; Who is involved in providing assistive products; How assistive products are provided; Considerations in assistive product provision (1 h).
3. Mobility Assistive Products: Mobility and different mobility problems; Mobility assistive products; How to carry out a mobility assistive products (3 h).
4. Walking Aids: Introduction; Step one: Select; Step two: Fit; Step three: Use 4; Step four: Follow up (4 h)
5. Transfer Boards: Introduction; Step one: Select; Step two: Fit; Step three: Use 4; Step four: Follow up (2 h), plus information about basic measurement of wheelchairs based on Wheelchair Service Training Package Basic Level (26)

In addition to the above, WHO's published pilot implementations in Fiji, Tajikistan and Papua New Guinea consistently adopted a blended format that combines e-learning with structured, supervised practical training. These pilots reported between 4 and 5 days of face-to-face skills training to consolidate the Select–Fit–Use–Follow-up competencies required for safe APs provision. Our study therefore followed this established WHO approach, adapting the format to the constraints and opportunities of Italian higher-education settings. Students were therefore engaged in 6 h of practical workshops and role plays focused on: (a) conducting mobility-assistive-product screening, and (b) hands-on application of the four-step protocol for walking aids (walking sticks, tri- and quadripods, elbow and axilla crutches, rollators and walking frames) and for transfer boards (with and without assistance) (c) take basic measurements of the body for wheelchair provision. While shorter than the practical components implemented in WHO's field pilots, these workshops were standardized, applied consistently across all cohorts, and necessary to ensure foundational competency within university timetable constraints.

A single facilitator – a senior Occupational Therapist (M.T.), experienced in AP provision – delivered all sessions to ensure consistency. Prior to teaching, the trainer completed the selected TAP modules online and obtained official WHO certification to guarantee familiarity with both content and pedagogical methodology. As the original TAP materials were available in English, UN languages and some other targeted languages (e.g., Ukrainian, Georgian, Swahili), all instructional content, including standardized checklists and screening forms, was translated into Italian by the trainer (M.T.) and subsequently reviewed by two institutional experts: a Medical Doctor in Physical and Rehabilitative Medicine (G.DB.) and an Associate Professor Neuro-Psychomotor Therapist (D.V.). This multi-stage translation and validation process ensured both linguistic accuracy and contextual relevance to Italian academic and clinical settings.

### Data collection and statistical analysis

Baseline demographic and academic data (age, gender, degree program) were collected via structured questionnaire and summarized using descriptive statistics (frequency, percentage, mean ± SD). To assess knowledge acquisition, a 56-item multiple-choice test was developed by adapting WHO TAP post-module quizzes and supplementing with context-specific items. The test comprised 30 items on “Introduction to APs” and “Mobility Assistive Products,” 15 on “Walking Aids,” and 11 on “Transfer Boards”. Each correct response earned one point; a subset of items allowed multiple correct options. Participants completed the test twice: immediately before the training (baseline) and immediately after completion of both theoretical and practical components (post-training). Both assessments were delivered through Google Forms, hosted within the university's institutional platform. Access required authenticated login through the university's password-protected system, and all data were stored securely in accordance with institutional data-protection and privacy regulations. Students were required to complete the test individually, in class, using their own smartphone, tablet, or laptop. For those without access to a personal device, equipment was provided by the university and/or the instructor. During test administration, students were supervised by the trainer. Only one attempt per student per assessment phase was permitted. To ensure data completeness and valid paired comparisons, only student who attended both the baseline and the post-training assessment were included in the analysis. Students who missed one of the assessments – due to absence or incomplete participation in the training – were excluded.

Data normality for all participants was evaluated using the Shapiro–Wilk test (27). Depending on distribution, pre-post differences in total knowledge scores were analyzed using paired Student's t-tests or Wilcoxon signed-rank tests. Effect sizes were calculated (Cohen's d for t-tests, r for nonparametric tests) to quantify the magnitude of knowledge gains (28). Pairwise comparisons between disciplinary groups were conducted using two-sided Mann–Whitney U tests to evaluate whether baseline and post-training score distributions differed significantly between academic programs (29). To control for multiple testing, *p*-values were Bonferroni-adjusted (*α* = 0.05/3 = 0.017). Statistical significance was set at *p* < 0.05. All analyses were performed using SPSS Statistics version 29.0 (IBM Corp., Armonk, NY, USA).

## Results

Training was conducted from May 2024 to June 2025. Of the 138 eligible participants, only those who completed both the pre- and post-training assessment were included in the analysis. Accordingly, we analyzed 105 completed responses sets for the Introduction module, 95 for the Mobility module, and 99 for the Transfer module. Socio-demographic characteristics of the participants for each module are summarized in Table 1.

**Table 1:** Socio-demographic characteristics of the participants.

Variables	TAP modules		
	Intro	Mobility	Transfer
Age mean (SD)	22.11 (2.86)	22.88 (3.12)	22.5 (3.16)
Sex	*N* (%)	*N* (%)	*N* (%)
Male	52 (49.53)	36 (37.89)	43 (43.43)
Female	53 (50.47)	59 (62.11)	56 (56.57)
Total	105 (100)	95 (100)	99 (100)
BSc	*N* (%)	*N* (%)	*N* (%)
PT	49 (46.67)	40 (42.11)	45 (45.46)
TNPEE	30 (28.57)	28 (29.47)	28 (28.28)
Nurses	26 (24.76)	27 (28.42)	26 (26.26)

We first assessed the distributional properties of the module-specific score data using the Shapiro–Wilk test. For the Introduction module (*n* = 105), the test statistic was W = 0.9304 (*p* < 0.001), indicating a significant departure from normality. Likewise, Mobility scores (*n* = 95) also violated the assumption of normality, with W = 0.9477 (*p* = 0.004), as well as the Transfer Boards dataset (*n* = 99) showed the greatest non-normality (W = 0.8689, *p* < 0.001). These results collectively confirmed that the score distributions for all three modules were non-normal, thereby justifying the subsequent use of nonparametric statistical tests.

All three TAP modules produced significant knowledge gains, each reaching statistical significance (*p* < 0.01) with large effect sizes. Results are summarized in Table 2.

**Table 2:** Pre- post-training scores of the three TAP modules.

TAP module	Participants	Pre-training median [IQR]	Post-training median [IQR]	Wilcoxon	*P* value	Effect size
Intro	105	23 [22–25]	26 [24.25–27]	624.5	<0.01	0.59
Mobility	95	9 [8–10]	12 [10–14]	385.6	<0.01	0.69
Transfers	99	6 [5–6]	7 [7–8]	133.5	<0.01	0.79

For Introduction Module, PT students showed a small, non-significant increase (*p* = 0.16; r = 0.34), whereas both TNPEE and Nursing cohorts achieved significant pre–post gains (TNPEE: *p* < 0.01, r = 0.81; Nursing: *p* < 0.01, r = 0.75), indicating large effect sizes for those groups. Pairwise tests confirm that no significant differences existed among cohorts either before or after training (all *p* > 0.05), suggesting that all programs started at similar baseline levels and ended with comparable post-training performance. Results are summarized in Table 3.

**Table 3:** Pre- post-training of Introduction module and pairwise comparison.

TAP INTRO MODULE	Pre-Training Median [IQR]	Post-Training Median [IQR]	*P* value	Effect size
PT	25.0 [23.0–26.0]	27.0 [25.0–28.0]	0.16	0.34
TNPEE	24.0 [22.0–26.0]	26.0 [24.0–27.0]	<0.01*	0.81
Nursing	24.5 [23.0–27.0]	26.0 [24.0–28.0]	<0.01*	0.75
Pairwise	Mann–Whitney U	P	Mann–Whitney U	P
PT vs. TNPEE	3,467.5	0.24	3,520.0	0.21
PT vs. Nurses	2,321.5	0.59	2,400.5	0.48
TNPEE vs. Nurses	1,875.0	0.16	1,925.0	0.28

*p < 0.05.

In Mobility module, both the PT and TNPEE groups demonstrated substantial, highly significant gains in mobility knowledge (*p* < 0.01; r = 0.72 and 0.80, respectively), whereas Nursing students showed a more modest, borderline improvement (*p* = 0.05; r = 0.45). Pairwise comparisons, PT and TNPEE did not differ pre- or post-training (*p* = 0.45 and 0.38). However, PT outperformed Nursing both before (U = 840; *p* = 0.01) and after training (U = 780; *p* = 0.012), and TNPEE also scored higher than Nursing post-training (U = 820; *p* = 0.015), indicating that the two rehabilitation cohorts gained more from the TAP mobility module than the Nursing cohort. Results are reported in Table 4.

**Table 4:** Pre- post-training of mobility module and pairwise comparison.

TAP MOBILITY MODULE	Pre-Training Median [IQR]	Post-Training Median [IQR]	*P* value	Effect size
PT	8.0 [7.0–9.0]	13.0 [11.0–14.0]	<0.01*	0.72
TNPEE	8.0 [7.0–9.0]	13.0 [12.0–14.0]	<0.01*	0.80
Nursing	9.0 [8.0–11.0]	11.0 [9.0–12.0]	0.05	0.45
Pairwise	Mann–Whitney U	P	Mann–Whitney U	P
PT vs. TNPEE	1,350.0	0.45	1,420.0	0.38
PT vs. Nurses	840.0	0.01 *	780.0	0.012 *
TNPEE vs. Nurses	900.0	0.05	820.0	0.015 *

*p < 0.05.

For Transfer Boards module all three cohorts showed significant knowledge gains post-training, with a *p* < 0.01 for both PT and TNPEE, with a r = 0.75 and 0.88, respectively, and Nursing student *p* = 0.01, r = 0.58. Pairwise tests indicate that none of the cohorts differed significantly from each other either before (all *p* ≥ 0.73) or after training (all *p* ≥ 0.72), confirming that the module produced comparable improvements across programs. Results are reported in Table 5.

**Table 5:** Pre- post-training of transfer boards module and pairwise comparison.

TAP TRANSFER MODULE	Pre-Training Median [IQR]	Post-Training Median [IQR]	*P* value	Effect size
PT	6.0 [4.0–6.0]	8.0 [7.0–9.0]	<0.01*	0.75
TNPEE	6.0 [5.0–6.0]	7.0 [7.0–8.0]	<0.01*	0.88
Nursing	6.0 [5.0–6.0]	8.0 [7.0–8.0]	0.01*	0.58
Pairwaise	Mann–Whitney U	P	Mann–Whitney U	P
PT vs. TNPEE	743.5	0.76	442.5	0.79
PT vs. Nurses	512.0	0.98	465.5	0.97
TNPEE vs. Nurses	264.5	0.73	270.5	0.72

*p < 0.05.

## Discussion

In this exploratory pre-post quasi experimental study, WHO's TAP was delivered in person to three cohorts of Italian undergraduate healthcare students with the aim of gauging knowledge acquisition across three core modules: an introductory overview of APs, mobility assistive devices, and transfer boards. Across all modules, students demonstrated statistically significant gains in median knowledge scores, confirming that modular training can substantially enhance competency in AT concepts and application.

The present study represents one of the first attempts to evaluate training in APs for undergraduate students of healthcare professionals. Training on wheelchair service provision is more explored at international landscape (30–33); however, educational initiatives on other APs remain limited (34). The first TAP pilot in Bangalore demonstrated high usability and engagement, with more than 60% of participants reporting that the e-learning platform was intuitive despite limited digital literacy. Furthermore, all modules – including those covering vision and walking aids – yielded substantial quiz score increases alongside rapid uptake of practical skills in hands-on sessions and role-plays (35). Subsequent TAP pilots in Papua New Guinea, Fiji, and Tajikistan further corroborated these results across diverse low- and middle-income country (LMIC) contexts (36–38). n Papua New Guinea, 19 primary care and prosthetics/orthotics personnel completed the modules during their work hours, reporting knowledge gains and increased confidence, but also emphasizing the importance of supervised practice to consolidate skills (38). In Fiji, 34 health workers—including nurses, physicians, physiotherapists, and community health staff—undertook a blended model combining e-learning, five days of in-person training, and three months of mentored service provision. This approach underscored the value of extended practical exposure and mentorship to translate theoretical knowledge into safe clinical practice (37). Similarly, in Tajikistan, 25 health workers completed TAP modules supplemented by four days of supervised training, with both participants and mentors recommending increased opportunities for practice and the inclusion of patients in role-play exercises (36). Taken together, these pilot experiences from LMICs demonstrate that TAP can enhance knowledge of APs and basic provision skills even in resource-constrained environments. At the same time, they consistently reveal that e-learning alone is insufficient: hands-on practice and mentoring remain indispensable for competency development.

Our study extends this evidence into a high-income country (HIC) context, assessing the integration of TAP within undergraduate curricula in Italy. Despite participants benefiting from higher baseline digital literacy and structured educational environments, outcomes were broadly consistent with LMIC pilots. We observed significant knowledge gains across all modules, with large effect sizes in both rehabilitation-focused (PT, TNPEE) and nursing cohorts, and high acceptability of TAP's structured four-step approach to AP provision. Discipline-specific patterns reflected curricular emphases: PT students, already familiar with mobility training, demonstrated smaller relative gains in introductory content but substantial improvements in mobility and transfer modules. By contrast, TNPEE and nursing students exhibited larger effect sizes in introductory content, reflecting their lower baseline exposure to AT.

The convergence of findings between LMIC pilots and this Italian study underscores TAP's adaptability across contexts. Whether implemented in settings with limited infrastructure or within structured university programs, TAP appears to provide a scalable and standardized foundation for developing AT competencies. At the same time, cross-context comparison highlights distinct implementation challenges. In LMICs, limited internet connectivity, lack of personal devices, and competing clinical duties were common barriers, whereas in the Italian setting institutional support and curricular integration facilitated feasibility. Nevertheless, a universal challenge emerges: across all contexts, learners consistently highlighted the necessity of structured opportunities for supervised practice.

From a health systems perspective, embedding TAP into Italian undergraduate curricula addresses persistent gaps in AT education identified in global or international studies (2, 39). Our findings indicate that TAP can complement existing coursework, standardize competencies across disciplines, and accelerate exposure to APs in programs where such content is traditionally underrepresented. Importantly, by adapting a tool originally designed for community health workers in LMICs, this study demonstrates TAP's relevance even in HICs, where shortages of rehabilitation professionals and uneven curricular coverage of AT remain pressing challenges.

While these pilots provide valuable insights into the feasibility of TAP in resource-limited contexts, much less is known about its applicability within structured higher-education settings in high-income countries. Addressing this gap, our study explored the integration of TAP into Italian undergraduate curricula.

In our study, the magnitude and consistency of gains varied by discipline and module. For the introductory module, TNPEE and nursing students exhibited very large effect sizes (r = 0.81 and 0.75, respectively) with *p* < 0.01, whereas PT students showed a medium, non-significant improvement (r = 0.34; *p* = 0.16). This pattern likely reflects PT students' comparatively higher baseline familiarity with APs, accrued through earlier integration of mobility practicums in their curriculum, which narrowed the measurable “learning window” on fundamental concepts (40). By contrast, TNPEE and nursing programs traditionally emphasize developmental motor skills or patient care fundamentals, leaving less curricular focus on assistive technology theory; thus, the TAP introduction module represented a more novel knowledge domain for these students.

The mobility module produced large, highly significant improvements in both PT and TNPEE cohorts (median rise from 8.0 to 13.0; r = 0.72 and 0.80; *p* < 0.01), reflecting both groups' engagement with practical device-selection and fitting protocols. PT students' early clinical placements introduce them to wheelchairs, crutches and walkers, which likely primed them to benefit maximally from TAP's structured four-step approach (Select, Fit, Use, Follow-up). TNPEE students, whose programs gradually introduce motor rehabilitation techniques, showed equally large gains, suggesting that TAP can expedite acquisition of device-specific competencies in disciplines where hands-on mobility training is emerging. Nursing students, by contrast, demonstrated a more modest increase (median 9.0 → 11.0; r = 0.45; *p* = 0.05), consistent with their more limited mobility-device exposure in clinical rotations focused on medical rather than rehabilitative care.

The transfer-boards module yielded the most uniform improvements across all three programs, PT (r = 0.75), TNPEE (r = 0.88) and nursing (r = 0.58), with a *p* ≤ 0.01, underscoring the universal value of standardized training in safe patient transfer techniques. This finding highlights that transfer skills (41), which span multiple care settings (acute, subacute, community), are perhaps under-emphasized in undergraduate curricula and benefit from focused, evidence-based instruction.

Between-group comparisons revealed few significant baseline or post-training differences, demonstrating that the modules were equally accessible and effective across diverse disciplinary backgrounds. This may also be explained by the fact that students were at the beginning of their education: both PT and TNPEE participants were in their first year of university, while nursing students were in their second year, which may have reduced differences across groups. An exception was the mobility module, where PT and TNPEE students outperformed nursing both before and after training (*p* < 0.02), reinforcing the notion that rehabilitative programs confer early, greater familiarity with mobility devices. However, by the end of training, absolute post-test medians converged, suggesting that TAP can level discipline-driven gaps in knowledge when properly integrated. A recent global environmental scan of mobility-assistive-product competencies identified 14 disparate standards and frameworks governing professional practice, yet revealed substantial inconsistencies in both the scope of competencies (e.g., assessment, prescription, fitting, follow-up) and the minimum educational level required across professions (42). These findings underscore a critical training gap: without a unified competencies framework, curricula remain unevenly aligned with service-delivery steps and often omit key domains of practice. Our results, showing variable baseline knowledge and learning gains among PT, TNPEE and nursing students, mirror this global heterogeneity and highlight TAP's potential to standardize both theoretical and practical competency development in mobility assistive products.

Despite these encouraging finding, the present study has several limitations to acknowledge. First, our Italian translation of TAP, though reviewed by clinical educators, was not an official WHO version and may differ subtly in nuance. Second, scheduling constraints led to the exclusion of students who completed only one of the two assessments, potentially introducing selection bias toward more motivated participants. Third, the absence of occupational therapy students and other disciplines (e.g., prosthetics and orthotics technicians) limits the generalizability of our conclusions across the full interprofessional spectrum. Fourth, although the selected WHO TAP modules are designed as a 10-hour online program, our university implementation followed WHO pilot practices by incorporating supervised practical sessions. The 6-hour duration of these workshops—while pedagogically appropriate—was adapted to the constraints of the academic timetable and may therefore differ from other TAP implementations, potentially limiting comparability across settings. In addition, the study did not include a standardized evaluation of practical skills or changes in clinical performance, highlighting the need for validated measures of hands-on competency in future research. Finally, the lack of published TAP evaluations in higher-education settings constrained our ability to benchmark effect sizes; additional studies are needed to establish standardized expectations.

A recent review underscore the persistent curricular gaps in AT education (43). Nonetheless, this study demonstrates that TAP is a feasible, high-impact educational intervention for undergraduate healthcare programs and can be considered as part of formal in-person training. Integrating TAP modules into existing curricula – especially for students whose training offers limited exposure to assistive products – can accelerate competency development, foster interprofessional understanding of AT ecosystems, and ultimately support more equitable access to safe, effective devices. Future work should aim to embed TAP in occupational therapy and interprofessional courses, evaluate long-term knowledge retention and translation into clinical practice, and explore blended-learning models that combine TAP's interactive e-learning with hands-on labs and simulated patient encounters. By doing so, health-education institutions can contribute meaningfully to WHO's GATE and Rehabilitation 2030 goals, preparing a workforce capable of advancing Universal Health Coverage through inclusive, evidence-based AT services.

## Data Availability

The original contributions presented in the study are included in the article/Supplementary Material, further inquiries can be directed to the corresponding author.
